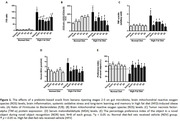# Prebiotic‐based snack from banana improves cognitive decline via decreased brain inflammation and brain oxidative stress in obese rats

**DOI:** 10.1002/alz.087199

**Published:** 2025-01-03

**Authors:** Piangkwan Sa‐nguanmoo, Nattayaporn Apaijai, Hiranya Pintana, Titikorn Chunchai, Busarin Arunsak, Sasiwan Kerdphoo, Wasana Pratchayasakul, Sakamon Devahastin, Nipon Chattipakorn, Siriporn C Chattipakorn

**Affiliations:** ^1^ Department of Physical Therapy, Faculty of Associated Medical Sciences, Chiang Mai University, Muang, Chiang Mai Thailand; ^2^ Neurophysiology Unit, Cardiac Electrophysiology Research and Training Center, Faculty of Medicine, Chiang Mai University, Chiang Mai Thailand; ^3^ Department of Physiology, Faculty of Medicine, Chiang Mai University, Chiang Mai Thailand; ^4^ Cardiac Electrophysiology Research and Training Center, Faculty of Medicine, Chiang Mai University, Chiang Mai Thailand; ^5^ Advanced Food Processing Research Laboratory, Department of Food Engineering, Faculty of Engineering, King Mongkut’s University of Technology Thonburi, Bangkok, Bangkok Thailand; ^6^ Department of Oral Biology and Diagnostic Sciences, Faculty of Dentistry, Chiang Mai University, Chiang Mai Thailand

## Abstract

**Background:**

Consuming prebiotics demonstrated therapeutic potential against obesity, as illustrated by our previous study on xylooligosaccharide (XOS), revealing that XOS reduced adiposity, diminished systemic inflammation, and restored cognitive function in obese insulin‐resistant rats through the gut‐brain axis. Fresh bananas at various ripening stages are being transformed into snacks, indicating potential as prebiotic‐based treats enriched with fructooligosaccharide and inulin. Despite those findings, there remains a notable gap in the literature concerning the impact of these prebiotic‐based snacks on brain inflammation, reactive oxygen species (ROS) production, and cognitive function in high‐fat diet (HFD)‐induced obese rats.

**Method:**

Seventy‐two male rats were divided into two groups, receiving either a normal diet (ND) or an HFD for total 16 weeks. At 13th week, ND‐fed rats were randomly assigned to 6 subgroups (n = 6/group) to receive either vehicle (NDV, 21 ml/kg/day), prebiotic‐based snacks prepared from bananas at ripening stages 2‐5 (NDS2‐5) (7.47 g dissolved in reversed osmosis water, 21 ml/kg/day in each subgroup), and inulin (NDI, 2 g/kg/day). Similarly, HFD‐fed rats were divided into 6 subgroups (n = 6/group): HFD‐fed rats receiving vehicle (HFV), prebiotic‐based snacks prepared from bananas at ripening stages 2‐5 (HFS2‐5) and inulin, with the same dose as ND‐fed rats, for 4 weeks. Cognitive function was assessed by novel object recognition (NOR). Serum malondialdehyde (MDA) levels, gut microbiota, brain inflammation, and brain ROS production were determined at the end of treatment.

**Result:**

The HFV group exhibited elevated serum MDA levels, increased brain mitochondrial ROS production, heightened TNF‐α levels, indicating brain inflammation, and an augmented *Firmicutes to Bacteroidetes (F/B)* ratio, indicating gut dysbiosis, when compared to the NDV group (p<0.05, **Figure 1**). Additionally, the HFV group showed cognitive decline, evidenced by a reduced percentage preference index in the NOR test (p<0.05, **Figure 1**). Remarkably, HFV rats receiving a prebiotic‐based snack showed reduced serum MDA levels, brain mitochondrial ROS production, brain inflammation, and improved gut dysbiosis, resulting in enhanced cognitive function. (p<0.05, **Figure 1**).

**Conclusion:**

A prebiotic‐based snack at all ripening stages holds promise as a neuroprotection against HFD‐induced obesity, offering multifaceted benefits by mitigating oxidative stress, gut dysbiosis, brain inflammation, and attenuated cognitive dysfunction in HFD‐fed rats.